# Care needs and preferences among older adults in an eastern Chinese province between 2013 and 2023: a repeated cross-sectional study

**DOI:** 10.3389/fpubh.2026.1939341

**Published:** 2026-10-08

**Authors:** Youyang Tang, Zhuojun Ye, Xiaoyue Wang, Xiaoyu Zhang, Yi Fan, Lingyun Liu, Zhousiqi Tang, Limei Jing

**Affiliations:** 1 School of Public Health, Shanghai Jiao Tong University School of Medicine, Shanghai, China; 2 Guangzhou Institute of Respiratory Health, the First Affiliated Hospital of Guangzhou Medical University, Guangzhou, China; 3 School of Public Health, Shanghai University of Traditional Chinese Medicine, Shanghai, China

**Keywords:** Andersen behavioral model, associated factors, care needs, care preferences, repeated cross-sectional study

## Abstract

**Background:**

Population aging and shrinking family structures are challenging traditional older adult care arrangements and widening the gap between older adults' care needs and available support. In China, policies promote integrated home, community, and institutional care. Understanding care needs, preferences, and associated factors is essential for developing evidence-informed care systems.

**Methods:**

We utilized data from an eastern Chinese province collected in the 2013, 2018, and 2023 waves of the China National Health Services Survey. Using the Andersen Behavioral Model as an exploratory conceptual framework, the independent variables were categorized into predisposing, enabling, and need factors. Wave-specific logistic regression models examined factors associated with care needs across all three waves and care preferences in 2018 and 2023. To examine the choice between traditional home care and formal community- or institution-based services, care preference was analyzed primarily as a binary outcome, while supplementary multinomial models retained all three original categories. Sequential blockwise logistic regression assessed the incremental contribution of each Andersen model dimension using pooled 2018 and 2023 data.

**Results:**

Our study included 10,934, 10,954, and 11,244 older adults in 2013, 2018, and 2023, respectively. Self-reported care needs remained stable at approximately 11%, whereas preferences for home care increased from 85.0% in 2018 to 93.0% in 2023. Need factors made the largest incremental contribution to care needs, while enabling and need factors made modest additional contributions to care preferences. Care needs were associated with age, health utility, chronic diseases, disability, dementia, sensory impairments, physical exercise, and social activity. Regarding care preferences, older age (2018: OR = 1.032; 2023: OR = 1.019) and being married were associated with a greater likelihood of preferring home care. Higher educational level was associated with community care (2018: OR = 1.150; 2023: OR = 1.356), whereas higher household income (2018: OR = 1.059; 2023: OR = 1.134), more chronic diseases (2018: OR = 1.097; 2023: OR = 1.127), and participation in social activity (2018: OR = 1.189; 2023: OR = 1.282) were associated with institutional care.

**Conclusions:**

Home care remained the predominant preference, while the prevalence of self-reported care needs showed little variation across the survey waves. Our findings underscore the importance of strengthening home-based support while maintaining affordable and accessible community- and institution-based care options.

## 1 Introduction

Rapid global population aging and shrinking family sizes are straining traditional older adult care arrangements and creating a significant gap between the growing needs of older adults and available support (1–3). Globally, many low- and middle-income countries face severe deficits in care provision (4), prompting international initiatives such as the WHO's Integrated Care for Older People (ICOPE), which emphasizes individualized, needs-based care (5). In China, traditional reliance on home care rooted in filial piety (6, 7), is being increasingly undermined by demographic transitions. In response, the government is fostering a multi-pillar system integrating home, community, and institutional care services to address the diverse needs of its aging population (8). A comprehensive understanding of older adults' care needs, preferences, and the factors associated with them is therefore critical to inform the design of effective, evidence-based older adult care systems.

Although extensive studies have utilized the Andersen Behavioral Model (9, 10) to explore the factors associated with older adults' care needs and preferences, the generalizability of many findings was potentially compromised by several common methodological limitations. Specifically, existing research were constrained by small sample sizes, self-developed questionnaires, and a limited scope of independent variables (11, 12). Furthermore, with the exception of two studies, previous research has focused on either care needs or preferences in isolation (13, 14). However, one such study used cross-sectional data from a single time point without capturing evolving care patterns over time (15). Another study, while considering multiple factors, did not employ a comprehensive theoretical framework like the Andersen Behavioral model to systematically analyze the full spectrum of associated factors (7).

To address these gaps, this study utilizes large-scale survey data collected in 2013, 2018, and 2023 from a province in eastern China. We first characterized cross-wave patterns in older adults' self-reported care needs across all three waves and in their care preferences in 2018 and 2023. Care preference was analyzed as a binary outcome to compare traditional home care with formal community and institutional care. A supplementary three-category analysis further distinguished factors associated with community and institutional care relative to home care. The Andersen Behavioral Model was used as an exploratory conceptual framework to organize potential correlates into predisposing, enabling, and need factors. Sequential blockwise logistic regression models based on the pooled 2018 and 2023 data evaluated the incremental contribution of each Andersen model dimension while wave-specific multivariable logistic regression models were used to examine the factors associated with care needs and preferences. Particular attention was given to disability, an important indicator of functional dependency (16), to examine whether its association with care preferences differed between the 2018 and 2023 survey waves. By integrating patterns in care needs and preferences with their associated factors, the findings provide context-specific evidence that may support the development of a more responsive and integrated older adult care system within the study province.

## 2 Methods

### 2.1 Data and sample

Data for this study were obtained from the provincial components of the 2013, 2018, and 2023 China National Health Services Survey (CNHSS) conducted in an eastern Chinese province. The CNHSS is a nationally representative survey conducted at 5-year intervals since 1993 by the Nation Health and Family Planning Commission of China (17). In the study province, each survey wave employed a multistage stratified cluster random sampling design covering all administrative districts. Households were selected through successive sampling of subdistricts, residential communities, and households. All sampled household members were invited to participate. Before participation, respondents were informed of the survey purpose and confidentiality arrangements, and written informed consent was obtained.

The study province is characterized by a relatively developed economy and an advanced stage of population aging (18). The CNHSS questionnaire comprised five sections covering household characteristics, individual household members, women of reproductive age, children, and older adults, with respondents completing the sections applicable to them. The present study included all respondents who were aged 60 years or older and completed the older-adult section, without further subsampling. The participant selection process is presented in Figure 1. After records with missing or logically inconsistent information were excluded, 33,132 older adults were included in the analysis: 10,934 in 2013, 10,954 in 2018, and 11,244 in 2023.

**Figure 1:**
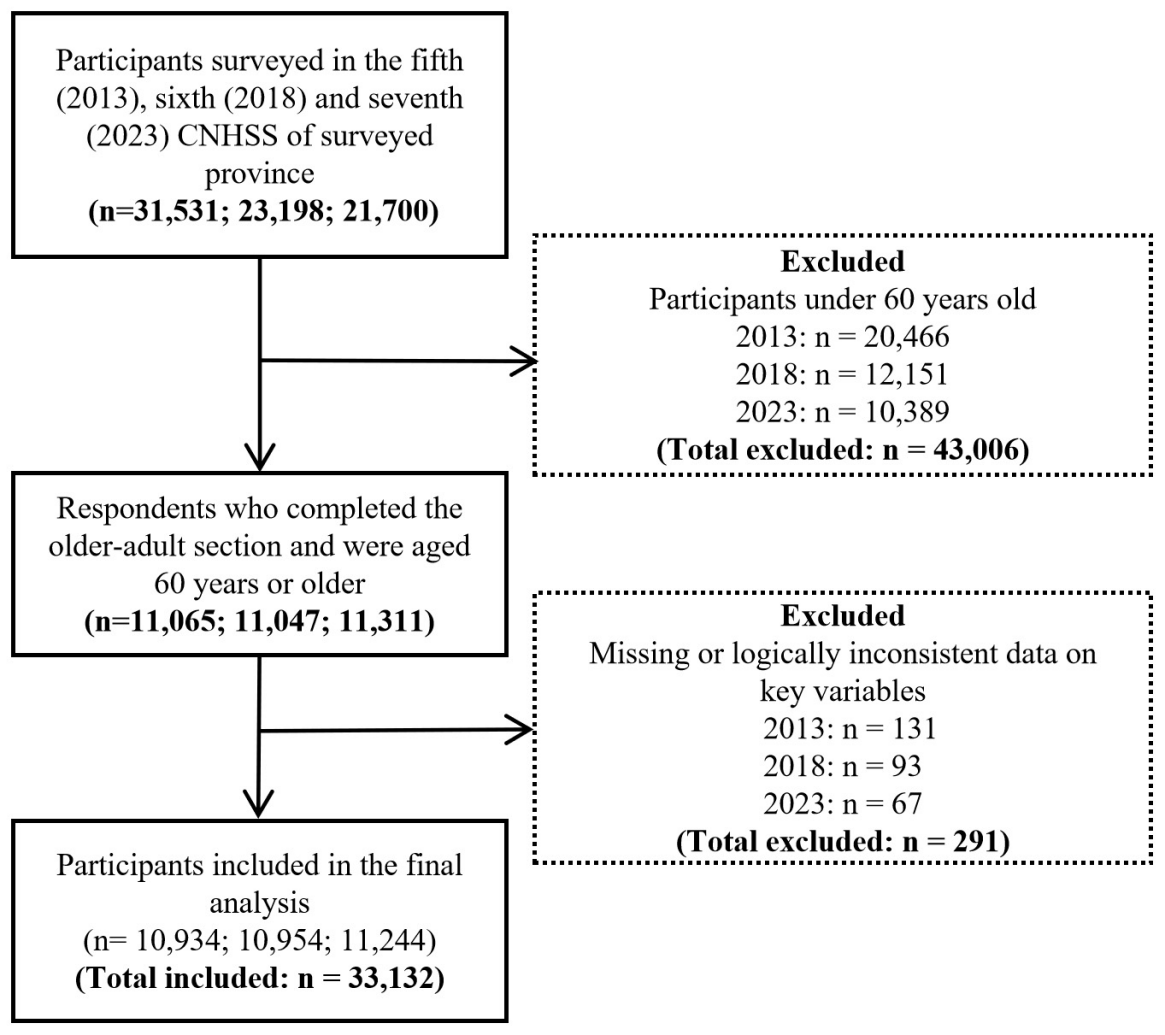
Flowchart of the study sample.

### 2.2 Variables

#### 2.2.1 Independent variables

The Andersen Behavioral Model was adopted as an exploratory conceptual framework for this study (19). This model classifies the factors related to healthcare utilization into three dimensions: predisposing, enabling, and need factors. The conceptual framework, including the variables examined in each dimension and the study outcomes, is presented in Supplementary Figure S1. Predisposing factors encompassed sociodemographic characteristics such as sex, age, marital status, and educational level. Enabling factors, representing economic or resource conditions, included social medical insurance and household income. Need factors, primarily reflecting health status and behaviors, comprised Body Mass Index (BMI), health utility value, disability, dementia, chronic diseases, smoking, drinking, physical exercise, health examination, hearing impairment, visual impairment, and social activity. Disability, dementia, and social activity were not assessed in the 2013 survey and were therefore included only in the analyses of the 2018 and 2023 data. All variables used in the final analysis are listed in Table 1, with selected variables further processed as described below.

**Table 1:** Sample characteristics.

Variables	2013	2018	2023	Test statistic^a^	*p*
	*N* = 10,934	*N* = 10,954	*N* = 11,244		
Predisposing factors					
**Sex (%)**				0.274	0.872
Men	5,180 (47.4)	5,154 (47.1)	5,295 (47.1)		
**Age [mean (SD)]**	70.39 (8.3)	70.08 (7.9)	71.04 (7.5)	42.960	< 0.001
**Marital status (%)**				165.158	< 0.001
Unmarried	154 (1.4)	102 (0.9)	123 (1.1)		
Married	8,620 (78.8)	9,041 (82.5)	9,259 (82.3)		
Widowed	2,026 (18.5)	1,634 (14.9)	1,575 (14.0)		
Divorced	130 (1.2)	160 (1.5)	259 (2.3)		
Other	4 (0.1)	17 (0.2)	28 (0.2)		
**Educational level (%)**				773.422	< 0.001
Primary school or below	4,771 (43.6)	3,588 (32.8)	3,064 (27.3)		
Junior High School	3,075 (28.1)	3,581 (32.7)	3,733 (33.2)		
Senior high/Technical/Vocational school	1,842 (16.8)	2,455 (22.4)	3,059 (27.2)		
College diploma or above	1,246 (11.4)	1,330 (12.1)	1,388 (12.3)		
Enabling factors					
**Social medical insurance (%)**				124.484	< 0.001
Yes	10,934 (100.0)	10,828 (98.8)	11,176 (99.4)		
**Household income (%)**				800.298	< 0.001
Low income	2,207 (20.2)	2,723 (24.9)	2,751 (24.5)		
Lower-middle income	2,495 (22.8)	1,978 (18.1)	2,466 (21.9)		
Middle income	1,927 (17.6)	3,020 (27.6)	2,527 (22.5)		
Upper-middle income	2,206 (20.2)	2,088 (19.1)	2,328 (20.7)		
High income	2,099 (19.2)	1,145 (10.5)	1,172 (10.4)		
Need factors					
**BMI (%)**				112.321	< 0.001
Underweight	825 (7.5)	700 (6.4)	549 (4.9)		
Normal	6,132 (56.1)	5,805 (53.0)	6,080 (54.1)		
Overweight	3,266 (29.9)	3,641 (33.2)	3,739 (33.3)		
Obesity	711 (6.5)	808 (7.4)	876 (7.8)		
**Health utility [Median (IQR)]**	0.96 (0.03)	0.97 (0.03)	0.96 (0.02)	6,639.901	< 0.001
**Chronic disease (%)**				145.145	< 0.001
0	4,066 (37.2)	3,722 (34.0)	3,503 (31.2)		
1	4,289 (39.2)	4,280 (39.1)	4,703 (41.8)		
≥2	2,579 (23.6)	2,952 (26.9)	3,038 (27.0)		
**Smoking (%)**				252.434	< 0.001
Yes/Once	2,076 (19.0)	2,907 (26.5)	3,069 (27.3)		
**Drinking (%)**				70.157	< 0.001
Yes	1,894 (17.3)	2,384 (21.8)	2,273 (20.2)		
**Exercise times weekly (%)**				379.667	< 0.001
0	4,947 (45.2)	4,255 (38.8)	3,935 (35.0)		
1~2	1,203 (11.0)	838 (7.7)	1,119 (10.0)		
≥3	4,784 (43.8)	5,861 (53.5)	6,190 (55.1)		
**Health examination (%)**				171.388	< 0.001
Yes	6,610 (60.5)	6,980 (63.7)	7,736 (68.8)		
**Hearing impairment (%)**				56.363	< 0.001
Easy to hear clearly	8,928 (81.7)	8,568 (78.2)	8,978 (79.8)		
Requires raised voice to hear clearly	1,503 (13.7)	1,700 (15.5)	1,724 (15.3)		
Difficult to hear clearly	503 (4.6)	686 (6.3)	542 (4.8)		
**Visual impairment (%)**				55.104	< 0.001
No/mild difficulty	9,102 (83.2)	8,815 (80.5)	9,390 (83.5)		
Moderate difficulty	1,538 (14.1)	1,745 (15.9)	1,472 (13.1)		
Extreme difficulty	294 (2.7)	394 (3.6)	382 (3.4)		
**Disability (%)** ^b^				3.110	0.375
Non-disability	NA	10,439 (95.3)	10,684 (95.0)		
Mild disability	NA	244 (2.2)	256 (2.3)		
Moderate disability	NA	104 (0.9)	100 (0.9)		
Severe disability	NA	167 (1.5)	204 (1.8)		
**Dementia (%)** ^b^				1.464	0.226
Yes	NA	161 (1.5)	144 (1.3)		
**Social activity (%)** ^b^				3,543.901	< 0.001
Yes	NA	2,338 (21.3)	6,824 (60.7)		
**Care needs (%)**				0.184	0.912
Yes	1,262 (11.5)	1,248 (11.4)	1,300 (11.6)		
**Care Preferences (%)** ^b^				374.484	< 0.001
Home care	NA	9,311 (85.0)	10,456 (93.0)		
Community care	NA	494 (4.5)	294 (2.6)		
Institutional care	NA	1,149 (10.5)	494 (4.4)		

NA, not assessed in the corresponding survey wave. ^a^Test statistics are Pearson's χ^2^ for categorical variables, one-way ANOVA F for approximately normally distributed continuous variables, and Kruskal–Wallis H for skewed continuous variables. ^b^Disability, dementia, social activity, and care preferences were not assessed in 2013; comparisons for these variables were therefore based on the 2018 and 2023 data.

For predisposing factors, educational level was categorized as primary school or below, junior high school, senior high/technical/vocational school and college diploma or above.

Enabling factors encompassed social medical insurance and household income. In 2013 and 2018, social medical insurance included the New Rural Cooperative Medical Scheme (NRCMS), Urban Resident Basic Medical Insurance (URBMI), Urban Employee Basic Medical Insurance (UEBMI), and other public health insurance programs. By 2023, NRCMS and URBMI had been consolidated into Urban and Rural Residents' Basic Medical Insurance (URRBMI). Household income was categorized into five groups, ranked from highest to lowest: high, upper-middle, middle, lower-middle, and low income.

In terms of need factors, BMI was classified into four categories: underweight (BMI < 18.5), normal weight (BMI 18.5~24.9), overweight (BMI 25.0~29.9), and obese (BMI ≥ 30.0). Health utility value, reflecting health-related quality of life, was measured using the EuroQol five dimensions questionnaire (EQ-5D) (20). The EQ-5D is a widely used multidimensional measure of health utility, covering five dimensions: mobility, self-care, usual activities, pain/discomfort, and anxiety/depression. The EQ-5D-3L (three-level version) was adopted in the 2013 and 2018 surveys, while the EQ-5D-5L (five-level version) was used in the 2023. To improve cross-wave comparability, the 2023 EQ-5D-5L responses were mapped onto the EQ-5D-3L utility scale (21), and all health states were valued using the same Chinese EQ-5D-3L value set (22). Higher values indicate better health-related quality of life. For the regression analyses, health utility was rescaled in 0.1-point increments so that the corresponding odds ratio represented the change in the odds of the outcome associated with a 0.1-point increase in health utility. Disability was assessed using the Katz Index of Independence in Activities of Daily Living (ADL), covering six basic functions: dressing, eating, bathing, transferring, toileting, and continence. Each item was rated on a four-point scale, and dichotomized into “independent” (no or some difficulty without assistance) and “dependent” (requiring assistance or unable to perform). Based on the number of dependent activities, disability was further classified as mild (dependent in 1~2 activities), moderate (3~4 activities), or severe (5~6 activities).

#### 2.2.2 Dependent variable

Care needs were assessed by asking, “In the past month, have you needed assistance with daily living?” with a binary response of “Yes” or “No.” This measure represents self-reported or perceived care needs in older adults. Care preferences were measured by the question, “Which type of older adult care do you prefer?” with response options: “Home care,” “Community care,” and “Institutional care.” The primary analysis was designed to examine the broad choice between traditional home care and the use of formal older adult care services delivered through community or institutional settings. Accordingly, community care and institutional care were combined into “community&institutional care,” for the binary logistic regression. To assess the robustness of the findings and distinguish factors associated with community and institutional care relative to home care, supplementary multinomial logistic regression analyses were conducted using the original three-category outcome, with home care as the reference category. Care preferences were not assessed in 2013 and was therefore analyzed only for the 2018 and 2023 waves.

### 2.3 Statistical analysis

Descriptive statistics were presented as means with standard deviations (SDs) for approximately normally distributed continuous variables, medians with interquartile ranges (IQRs) for skewed continuous variables, and frequencies with percentages for categorical variables. Differences in participant characteristics across survey waves were assessed using Pearson's chi-square tests for categorical variables, one-way analysis of variance for approximately normally distributed continuous variables, and the Kruskal-Wallis test for skewed continuous variables.

Guided by the Andersen Behavioral Model, sequential blockwise logistic regression models were constructed to evaluate the incremental contributions of predisposing, enabling, and need factors to care needs and care preferences. Given the availability of variables across survey waves, we pooled the 2018 and 2023 data and included survey year as a dummy variable. Model 1 included survey year and predisposing factors; Model 2 additionally incorporated enabling factors; and Model 3 further included need factors. The incremental contribution of each block was evaluated using block likelihood-ratio chi-square tests and changes in Nagelkerke's R^2^ (23). To formally assess whether the association between disability severity and care preferences differed between the two survey waves, a survey year × disability interaction term was added to the fully adjusted pooled model (24).

Multivariable binary logistic regression models were fitted to estimate factors associated with the two outcomes in each survey wave. Care needs were analyzed separately for 2013, 2018, and 2023, whereas care preferences were analyzed for 2018 and 2023. To retain the distinction among the three original care preferences categories, supplementary multinomial logistic regression models were fitted, using home care as the reference outcome. The supplementary models included the same conceptual set of independent variables as the primary models, with marital status grouped into two categories (married/not married) to reduce sparse cells across the three outcome categories. All predisposing, enabling, and need factors available in each corresponding survey wave were entered simultaneously into models. Multicollinearity among the included independent variables was assessed using variance inflation factors (VIFs) and tolerance values to ensure the stability of the model estimates. VIFs >5 or tolerance values < 0.20 were considered indicative of potentially concerning multicollinearity (25). As individual sampling weights and the original stratum and primary sampling unit identifiers were not available in the provincial analytic datasets, the analyses were conducted using conventional procedures without survey weighting. Accordingly, the estimates are interpreted primarily as associations observed within the analyzed samples, and their extension to the broader population should be approached with appropriate caution. All analyses were performed using RStudio 4.4.1 and IBM SPSS Statistics 25, with two-tailed *p*-values < 0.05 considered statistically significant.

## 3 Results

### 3.1 Sample characteristics

As for the sociodemographic characteristics of respondents, data from the three survey waves consistently indicated that approximately 47% of them were men, while the average age significantly increased from 70.39 to 71.04 years between 2013 and 2023 (*p* < 0.001). The majority of respondents were married, comprising around 80% of the sample, followed by widowed individuals. Educational level improved significantly, with about 70% of respondents attaining at least a junior high school education by 2023 (*p* < 0.001). The proportion of respondents classified as middle-income increased, from 17.6% in 2013 to 22.5% in 2023 (*p* < 0.001). With regard to health-related factors, the prevalence of overweight and obesity rose substantially, with nearly 40% of respondents categorized as overweight or obesity in 2023 (*p* < 0.001). Health utility values exhibited minor fluctuations across the three survey periods but remained consistently above 0.96. The prevalence of chronic diseases increased notably, with the percentage of respondents without any chronic diseases declining from 37.2% in 2013 to 31.2% in 2023 (*p* < 0.001). The proportions of older adults with smoking and drinking were higher in 2023 compared to 2013 (*p* < 0.001). Approximately 20% of respondents reported experiencing mild or greater hearing or visual impairment. While no statistically significant disparities were observed, the proportion of older individuals with disability in 2023 exhibited a slight increase, rising from 4.7% in 2018 to 5.0% in 2023, whereas the prevalence of dementia declined marginally from 1.5% in 2018 to 1.3% in 2023. In terms of factors about health behavioral, physical exercise inactivity decreased markedly, with the proportion of respondents who never engaged in exercise dropping from 45.2% in 2013 to 35.0% in 2023 (*p* < 0.001). The percentage of respondents who had received a health examination within the past 12 months demonstrated a steady upward trend, increasing from 60.5% in 2013 to 63.7% in 2018 and further to 68.8% in 2023 (*p* < 0.001). The proportion of older adults participating in social activities increased from 21.3% in 2018 to 60.7% in 2023 (*p* < 0.001) (Table 1).

### 3.2 Care needs and preferences

The proportion of older adults (aged 60 and above) reporting care needs remained relatively stable between 2013 and 2023, consistently around 11% (Table 1). Women reported a significantly higher prevalence of care needs than men in each survey wave (2023: 12.24% vs. 10.80%, *p* = 0.018). Additionally, care needs increased markedly with age (*p* < 0.001), reaching 37.94% among those aged 80 and above in 2023 (Tables 2, 3).

**Table 2:** Comparison of care needs, preferences by sex in 2013, 2018, 2023.

Variables	2013				2018				2023			
	Women	Men	χ^2^	*p*	Women	Men	χ^2^	*p*	Women	Men	χ^2^	*p*
**Care needs (%)**	12.72	10.23	16.553	< 0.001	12.41	10.24	12.722	< 0.001	12.24	10.80	5.640	0.018
**Home Care preferences (%)** ^**a**^	NA	NA	NA	NA	84.71	85.33	0.836	0.361	92.79	93.22	0.800	0.371

NA, not assessed in the corresponding survey wave. ^a^Care preferences were not assessed in 2013; therefore, no estimates or corresponding within-wave comparison *p*-values are reported for that wave.

**Table 3:** Comparison of care needs, preferences by age group in 2013, 2018, 2023.

Variables	2013					2018					2023				
	60–69	70–79	≥80	χ^2^	*p*	60–69	70–79	≥80	χ^2^	*p*	60–69	70–79	≥80	χ^2^	*p*
**Care needs (%)**	5.11	11.87	31.44	984.247	< 0.001	5.75	11.24	33.40	960.832	< 0.001	5.11	10.41	37.94	1,255.490	< 0.001
**Home Care preferences (%)** ^**a**^	NA	NA	NA	NA	NA	83.43	85.78	89.49	38.784	< 0.001	92.36	93.46	93.92	6.765	0.034

NA, not assessed in the corresponding survey wave. ^a^Care preferences were not assessed in 2013; therefore, no estimates or corresponding within-wave comparison *p*-values are reported for that wave.

Regarding care preferences, home care remained the predominant choice, increasing from 85.0% in 2018 to 93.0% in 2023, while preferences for community care decreased from 4.5% to 2.6% and those for institutional care decreased from 10.5% to 4.4% (*p* < 0.001) (Table 1). Although no statistically significant sexual disparities in home care preference were found, the proportion of older men opting for home care was higher than that of older women. Notable age-related differences were observed (*p* < 0.05), with 93.92% of those aged 80 and above opting for home care in 2023 (Tables 2, 3).

### 3.3 Factors associated with care needs

Sequential blockwise analyses demonstrated the incremental contributions of the three Andersen model dimensions to care needs. Predisposing factors yielded an initial Nagelkerke R^2^of 0.180, enabling factors produced only a small increment, and need factors made the largest additional contribution, increasing Nagelkerke R^2^to 0.515. Each block significantly improved model fit (all *p* < 0.001; Supplementary Table S1).

Across the three wave-specific models, several predisposing and need factors were associated with care needs. Among predisposing factors, advancing age was consistently associated with increased likelihood of care needs (2013: OR = 1.038; 2018: OR = 1.032; 2023: OR = 1.045). Regarding need factors, a greater number of chronic diseases (2013: OR = 1.190; 2018: OR = 1.244; 2023: OR = 1.344), and more severe hearing impairment (2013: OR = 1.606; 2018: OR = 1.170; 2023: OR = 1.583) and more severe visual impairment (2013: OR = 1.603; 2018: OR = 1.346; 2023: OR = 1.240) significantly increased the odds of needing care. In contrast, each 0.1-point increase in health utility (2013: OR = 0.276; 2018: OR = 0.312; 2023: OR = 0.268) and more frequent weekly physical exercise (2013: OR = 0.843; 2018: OR = 0.860; 2023: OR = 0.838) were associated with lower odds of reporting care needs (Table 4).

**Table 4:** Multivariable logistic regression models for care needs in 2013, 2018, and 2023.

Variables	2013			2018			2023		
	OR (95%CI)	SE	*p*	OR (95%CI)	SE	*p*	OR (95%CI)	SE	*p*
Predisposing factors									
Men (ref: Women)	1.135 (0.948, 1.358)	0.092	0.167	1.247 (1.013, 1.536)	0.106	0.037	1.035 (0.824, 1.300)	0.116	0.766
Age	1.038 (1.027, 1.048)	0.005	< 0.001	1.032 (1.021, 1.044)	0.006	< 0.001	1.045 (1.033, 1.058)	0.006	< 0.001
Unmarried (ref: Married)	1.133 (0.625, 2.057)	0.304	0.681	1.361 (0.656, 2.825)	0.373	0.408	2.285 (1.149, 4.546)	0.351	0.019
Widowed	1.224 (1.010, 1.484)	0.098	0.039	1.399 (1.137, 1.722)	0.106	0.002	1.242 (0.993, 1.555)	0.114	0.058
Divorced	0.665 (0.290, 1.525)	0.424	0.335	0.531 (0.206, 1.370)	0.483	0.190	1.230 (0.691, 2.192)	0.295	0.481
Other^a^	9.751 (0.932, 101.975)	1.198	0.057	3.371 (0.745, 15.253)	0.770	0.115	0.010 (0.001, 510,412.813)	9.041	0.613
Educational level	1.047 (0.960, 1.143)	0.044	0.297	0.931 (0.849, 1.021)	0.047	0.127	1.001 (0.909, 1.102)	0.049	0.991
Enabling factors									
Social medical insurance (ref: No)	NE	NE	NE	2.262 (0.869, 5.886)	0.488	0.094	3.624 (0.720, 18.251)	0.825	0.118
Household income	1.054 (0.992, 1.119)	0.031	0.091	0.975 (0.910, 1.045)	0.035	0.476	0.990 (0.920, 1.065)	0.037	0.782
Need factors									
Underweight (ref: Normal weight)	0.946 (0.732, 1.222)	0.131	0.670	0.912 (0.687, 1.212)	0.145	0.526	1.187 (0.858, 1.640)	0.165	0.300
Overweight	0.885 (0.740, 1.058)	0.091	0.180	0.754 (0.629, 0.905)	0.093	0.002	0.819 (0.677, 0.989)	0.097	0.038
Obesity	0.804 (0.589, 1.098)	0.159	0.170	0.981 (0.739, 1.303)	0.145	0.894	0.861 (0.629, 1.177)	0.160	0.348
Health utility	0.276 (0.249, 0.305)	0.052	< 0.001	0.312 (0.277, 0.351)	0.060	< 0.001	0.268 (0.234, 0.307)	0.069	< 0.001
Chronic disease	1.190 (1.106, 1.282)	0.038	< 0.001	1.244 (1.157, 1.338)	0.037	< 0.001	1.344 (1.237, 1.460)	0.042	< 0.001
Smoking (ref: No)	0.870 (0.683, 1.108)	0.123	0.258	0.851 (0.668, 1.084)	0.124	0.191	1.173 (0.910, 1.512)	0.130	0.219
Drinking (ref: No)	0.803 (0.625, 1.033)	0.128	0.087	0.786 (0.620, 0.996)	0.121	0.046	0.624 (0.480, 0.810)	0.134	< 0.001
Physical exercise	0.843 (0.773, 0.919)	0.044	< 0.001	0.860 (0.790, 0.936)	0.043	0.001	0.838 (0.765, 0.918)	0.046	< 0.001
Health examination (ref: No)	0.927 (0.795, 1.080)	0.078	0.328	0.902 (0.768, 1.060)	0.082	0.211	0.858 (0.720, 1.022)	0.089	0.087
Hearing impairment	1.606 (1.417, 1.820)	0.064	< 0.001	1.170 (1.029, 1.330)	0.065	0.016	1.583 (1.380, 1.817)	0.070	< 0.001
Visual impairment	1.603 (1.400, 1.835)	0.069	< 0.001	1.346 (1.173, 1.545)	0.070	< 0.001	1.240 (1.069, 1.439)	0.076	0.005
Disability	NA	NA	NA	5.709 (4.207, 7.746)	0.156	< 0.001	6.662 (4.664, 9.516)	0.182	< 0.001
Dementia (ref: No)	NA	NA	NA	2.270 (1.310, 3.936)	0.281	0.003	6.674 (3.579, 12.446)	0.318	< 0.001
Social activity (ref: No)	NA	NA	NA	0.686 (0.542, 0.868)	0.120	0.002	0.389 (0.326, 0.464)	0.090	< 0.001

NE, not estimable. The regression coefficient for social medical insurance in 2013 could not be estimated because all respondents in the analytic sample reported insurance coverage, resulting in no variation in this variable. ^a^The “Other” marital-status category contained few participants across the survey waves (2013: *n* = 4; 2018: *n* = 17; 2023: *n* = 28), resulting in sparse data and imprecise estimates. The corresponding odds ratios and confidence intervals should therefore be interpreted with caution. SE, standard error of the regression coefficient; OR, adjusted odds ratio; CI, confidence interval.

Among the variables assessed only in 2018 and 2023, greater disability severity was associated with higher odds of reporting care needs (2018: OR = 5.709; 2023: OR = 6.662). Similarly, dementia was associated with higher odds of requiring care (2018: OR = 2.270; 2023: OR = 6.674). In contrast, participation in social activity was associated with reduced care needs (2018: OR = 0.686; 2023: OR = 0.389) (Table 4).

No substantial multicollinearity was identified in the models for care needs, with all VIFs below 3 and all tolerance values exceeding 0.20 (Supplementary Table S2).

### 3.4 Factors associated with care preferences

In the blockwise analysis, predisposing factors yielded an initial Nagelkerke R^2^ of 0.050, the additional contributions of

enabling and need factors were comparatively modest, increasing Nagelkerke R^2^ to 0.051 and 0.057, respectively. Nevertheless, each block significantly improved model fit (all *p* < 0.001; Supplementary Table S1).

In the binary logistic regression and supplementary multinomial analyses, several predisposing, enabling, and need factors were associated with older adults' care preferences. Among predisposing factors, older age was associated with stronger preferences for home care (2018: OR = 1.032; 2023: OR = 1.019). Compared to married individuals, those who were unmarried (2018: OR = 0.443; 2023: OR = 0.272), widowed (2018: OR = 0.818; 2023: OR = 0.629) and divorced (2018: OR = 0.612; 2023: OR = 0.458) had lower odds of preferring home care. Higher educational level was consistently associated with greater odds of preferring community care over home care (2018: OR = 1.150; 2023: OR = 1.356). For enabling factors, higher household income was linked to a greater likelihood of preferring institutional care relative to home care (2018: OR = 1.059; 2023: OR = 1.134). Regarding need factors, a greater number of chronic diseases (2018: OR = 1.097; 2023: OR = 1.127), and participation in social activity (2018: OR = 1.189; 2023: OR = 1.282) were associated with institutional care relative to home care. Full results are presented in Table 5; Supplementary Tables S4, S5.

**Table 5:** Multivariable logistic regression models for care preferences in 2018, 2023.

Variables	2018			2023		
	OR (95%CI)	SE	*p*	OR (95%CI)	SE	*p*
Predisposing factors						
Men (ref: Women)	1.122 (0.968, 1.300)	0.075	0.127	1.193 (0.968, 1.470)	0.107	0.098
Age	1.032 (1.024, 1.041)	0.004	< 0.001	1.019 (1.007, 1.031)	0.006	0.002
Unmarried (ref: Married)	0.443 (0.286, 0.686)	0.223	< 0.001	0.272 (0.175, 0.424)	0.226	< 0.001
Widowed	0.818 (0.691, 0.969)	0.086	0.020	0.629 (0.505, 0.783)	0.112	< 0.001
Divorced	0.612 (0.423, 0.886)	0.189	0.009	0.458 (0.317, 0.661)	0.187	< 0.001
Other^a^	3.040 (0.401, 23.059)	1.034	0.282	2.283 (0.309, 16.881)	1.021	0.419
Educational level	0.900 (0.846, 0.957)	0.032	0.001	0.879 (0.806, 0.959)	0.044	0.004
Enabling factors						
Social medical insurance (ref: No)	0.749 (0.428, 1.313)	0.286	0.313	0.885 (0.318, 2.463)	0.522	0.815
Household income	0.931 (0.889, 0.976)	0.024	0.003	0.907 (0.852, 0.966)	0.032	0.002
Need factors						
Underweight (ref: Normal weight)	0.800 (0.642, 0.996)	0.112	0.046	0.881 (0.629, 1.233)	0.172	0.460
Overweight	1.141 (1.013, 1.286)	0.061	0.030	1.095 (0.929, 1.290)	0.084	0.280
Obesity	1.130 (0.915, 1.397)	0.108	0.257	1.005 (0.762, 1.326)	0.141	0.971
Health utility	1.117 (1.012, 1.232)	0.050	0.027	1.120 (0.981, 1.280)	0.068	0.094
Chronic disease	0.901 (0.852, 0.951)	0.028	< 0.001	0.892 (0.824, 0.966)	0.041	0.005
Smoking (ref: No)	0.987 (0.837, 1.163)	0.084	0.872	0.778 (0.620, 0.976)	0.116	0.030
Drinking (ref: No)	0.865 (0.745, 1.004)	0.076	0.057	1.025 (0.833, 1.259)	0.106	0.819
Physical exercise	0.941 (0.886, 1.000)	0.031	0.048	1.005 (0.924, 1.093)	0.043	0.911
Health examination (ref: No)	1.070 (0.958, 1.197)	0.057	0.231	0.959 (0.815, 1.128)	0.083	0.612
Hearing impairment	0.951 (0.860, 1.051)	0.051	0.326	1.111 (0.944, 1.307)	0.083	0.205
Visual impairment	1.003 (0.893, 1.126)	0.059	0.963	0.912 (0.774, 1.074)	0.084	0.271
Disability	1.327 (1.068, 1.650)	0.111	0.011	1.044 (0.822, 1.326)	0.122	0.725
Dementia (ref: No)	0.587 (0.380, 0.908)	0.222	0.017	0.694 (0.377, 1.277)	0.312	0.240
Social activity (ref: No)	0.839 (0.739, 0.952)	0.065	0.007	0.786 (0.668, 0.925)	0.083	0.004

^a^The “Other” marital-status category included few respondents in the survey waves analyzed for care preferences (2018: *n* = 17; 2023: *n* = 28), resulting in sparse data and imprecise estimates. The corresponding odds ratios and confidence intervals should therefore be interpreted with caution. SE, standard error of the regression coefficient; OR, adjusted odds ratio; CI, confidence interval.

Diagnostic analyses further showed that the models for care preferences had no substantial multicollinearity, with all VIFs were below 3 and all tolerance values exceeded 0.20 (Supplementary Table S3).

Greater disability severity was associated with higher odds of preferring home care in 2018 (OR = 1.327, *p* = 0.011), whereas no statistically significant association was observed in 2023 (OR = 1.044, *p* = 0.725). The pooled analysis further identified a significant survey year × disability interaction (OR = 0.752, *p* = 0.007), indicating that the association between disability severity and home care preference was weaker in 2023 than in 2018 (Supplementary Table S6).

## 4 Discussion

This repeated cross-sectional study examined patterns in self-reported care needs across the 2013, 2018, and 2023 survey waves and in care preferences in 2018 and 2023 among adults aged 60 years or older in an eastern Chinese province. The Andersen Behavioral Model was used as an exploratory framework to organize the associated factors into predisposing, enabling, and need dimensions. We evaluated the incremental contributions of these three dimensions using the pooled 2018 and 2023 data, and further examined factors associated with care needs and preferences in the wave-specific analyses. Our findings provide exploratory, context-specific evidence to support the planning and development of older adult care services that better address the diverse needs and preferences of older adults within the study province.

Within the exploratory application of the Andersen Behavioral Model, our findings indicated the three dimensions showed different incremental contributions to the two outcomes. Need factors produced the largest incremental improvement in the model for care needs, highlighting the close relationship between perceived care needs and health and functional status. By contrast, enabling and need factors produced relatively modest increments in the model for care preferences. This finding suggests that care preferences may also be shaped by factors not fully captured in the available survey data, such as family caregiving capacity, service availability, cultural expectations, and previous care experiences. Given the repeated cross-sectional design and the exploratory scope of the study, dimension-level interactions, mediation pathways, and moderation mechanisms were not examined.

Improvements in health behaviors coexisted with an increasing burden of age-related health conditions. We observed notable increases in the proportions of older adults engaging in physical exercise, receiving health examinations, and participating in social activity. These changes may partly reflect broader efforts to strengthen health education and health promotion among older adults (26). However, we observed increases in key health indicators including the prevalence of chronic diseases, hearing impairments, and disability among the older adults across the survey waves, consistent with prior studies (27). These patterns may partly relate to population aging (28, 29), which is more vulnerable to multiple health conditions (30). The coexistence of improved health behaviors and a growing burden of health conditions suggests that behavioral improvements may not translate immediately into observable improvements in health status within the surveyed samples (31). Sustained, long-term interventions are therefore needed to promote healthy aging and reduce the burden of age-related health conditions.

Our study found that the proportion of older adults requiring care remained relatively stable, with home care consistently emerging as the predominant preference. The stability of reported care needs may reflect the combined influence of population aging and broader improvements in health and care support, although these underlying mechanisms were not directly examined in this study (32). Additionally, the increasing availability of assistive living technologies (33) may help some older adults with functional limitations maintain independence and continue living at home. The enduring preference for home care aligns with previous findings in China (10, 34), likely shaped by traditional cultural values such as filial piety and familism, which emphasizes family support in familiar environments (35). Government initiatives promoting integrated home and community care models may also contribute to the feasibility of aging in place (36). Internationally, similar patterns suggest that home care remains a globally preferred option (7). These insights highlight the importance of developing a diversified older adult care system that strengthens home-based support, expands accessible community services to facilitate aging in place, and preserves affordable institutional care for older adults requiring more intensive or continuous assistance.

Older women exhibited higher needs for care but lower preferences for home care compared to older men. This disparity may be related to women's generally poorer self-reported health status compared to men (37, 38), and their greater willingness to acknowledge and articulate health concerns and care needs (39, 40). In contrast, their lower preference for home care may be associated with social and demographic realities (41). Older women generally have longer life expectancies and higher rates of widowhood, are significantly more likely to live alone (42). For this expanding demographic, traditional family-based support may be limited (43). Community-based services may supplement reduced family support while allowing older women to continue living in familiar neighborhoods, whereas institutional care may be more appropriate when sustained assistance, supervision, or medical support is required. Policies should identify unmet care needs that may be masked by low expressed preference among older men, while providing older women with tiered options ranging from community-based support to affordable institutional care according to the intensity of their needs and the availability of informal care.

Among predisposing factors, advanced age was positively associated with both greater care needs and a stronger preference for home care. This may reflect greater health burdens among older individuals and their emotional attachment to and sense of security within familiar environments (44, 45). However, given mixed evidence on the relationship between age and home care preferences (46, 47), further research is needed. Higher educational level was associated with greater acceptance of community care, aligning with existing research (48). Furthermore, marital status was associated with care preferences. Married individuals were more likely to prefer home care, possibly due to the availability of support from spouses or children, whereas unmarried older adults tended to prefer community or institutional care (49).

As for enabling factors, higher household income was associated with higher odds of preferring institutional care. Given the higher costs of institutional care, older adults with lower household income were more inclined to choose home care as a cost-saving option (50). Household income was not significantly associated with care needs, suggesting that perceived need for assistance may be more closely related to health conditions and functional limitations than to financial resources. Additionally, participation in social medical insurance did not show a significant association with either care needs or preferences. This may be attributed to the broad and relatively uniform coverage provided by China's social medical insurance system, which helps mitigate disparities in care access (51). To ensure care preferences reflect genuine needs, not economic limitations, policies should improve affordability through tiered subsidies and continued development of long-term care financing arrangements.

In terms of need factors, while various health issues were consistently linked to greater care needs (52, 53), their relationships with care preferences varied. A greater number of chronic diseases was associated with a higher preference for institutional care over home care. This association may reflect the cumulative complexity of multimorbidity rather than the effect of any single condition. Chronic diseases may be accompanied by interacting physiological and psychological burdens, including oxidative imbalance and depression, aging-related cellular senescence and chronic inflammation, as well as immune dysfunction and increased susceptibility to infection (54–56). These overlapping problems can contribute to functional decline, complex medication regimens, frequent clinical monitoring, and an increased risk of acute complications, thereby placing substantial demands on patients and family caregivers. When such care demands are perceived to exceed the capacity of individuals or families to manage them at home, older adults may consider institutional settings that provide more continuous supervision, professional care, and access to medical support. By contrast, hearing and visual impairment showed no significant associations with care preferences, possibly because sensory impairments alone may not necessitate a change in care setting, particularly when their effects can be managed through assistive devices and environmental adaptations. Their relevance to care preferences may also depend on the extent to which they result in broader functional limitations or dependence. Accordingly, care preferences may be more closely related to overall functional capacity, the complexity of required care, and the availability of support than to the presence of a specific health condition in isolation.

Our analysis also identified significant associations of health behaviors with care needs and preferences. Frequent physical exercise and social activity participation were associated with lower care needs, which may be consistent with the potential benefits of these activities for physical and cognitive functioning (57), although the cross-sectional design does not establish the direction of these relationships. Interestingly, social activity was associated with lower care needs and a greater preference for institutional care. One possible explanation is that these individuals may place greater value on opportunities for continued social engagement, which they may perceive as more readily available in institutional settings than in isolated home environments (58). In this context, the preference for institutional care appears to reflect proactive social motivations rather than responses to functional decline.

Greater disability severity was significantly associated with a stronger preference for home care in 2018, whereas the association was weaker and no longer statistically significant in 2023. This pattern suggests that care preferences among older adults with disability may be related not only to the severity of disability but also to the accessibility, affordability, and perceived quality of professional care services (59), as well as older adults' acceptance of different care models (60). Continued investment in differentiated care options may therefore help accommodate their diverse needs and preferences. Accessible community-based services can provide rehabilitation, respite care, and daily-living support close to home, whereas affordable institutional care is needed for older adults requiring sustained supervision or more intensive assistance.

Several limitations should be considered when interpreting these findings. First, although a comprehensive set of variables was selected according to the Andersen Behavioral Model, other relevant factors may not have been captured, and residual confounding cannot be excluded. Second, several measures in the CNHSS were self-reported and may therefore be subject to reporting or recall bias. In addition, care needs were assessed using a single item that captures perceived need for assistance rather than the severity or multiple dimensions of objectively assessed functional dependency. Third, the analysis was restricted to an eastern Chinese province, and sampling weights and the original stratum and primary sampling unit identifiers were unavailable. Consequently, the estimates reflect associations within the analyzed samples; standard errors may have been underestimated, and nominally significant findings and their broader generalizability should be interpreted cautiously. Finally, given the observational and repeated cross-sectional design, the findings demonstrate associations rather than causal relationships. Future longitudinal studies are needed to further clarify the temporal relationships between the identified factors and older adults' care needs and preferences and to provide stronger evidence for causal inference.

## 5 Conclusion

Home care was the predominant care preference in both 2018 and 2023 among older adults surveyed in an eastern Chinese province, while the prevalence of self-reported care need remained relatively stable across the three survey waves. The analysis indicated that care needs were mainly associated with predisposing and need factors, with need factors producing the largest incremental improvement in model fit. Care preferences were associated with factors across the predisposing, enabling, and need dimensions, although the incremental contributions of enabling and need factors were comparatively modest. The distinct patterns associated with home, community, and institutional care underscore the need for differentiated service planning, including strengthening home-based support, expanding accessible community services that facilitate aging in place, and maintaining affordable institutional care for older adults requiring more intensive or continuous support. Future longitudinal and multi-regional studies are warranted to further characterize changes in older adults' care needs and preferences and clarify the factors associated with their evolving care patterns.

## Data Availability

The data analyzed in this study is subject to the following licenses/restrictions: The data that support the findings of this study can be provided by the corresponding author, upon reasonable request. Requests to access these datasets should be directed to LJ, limei_jing@163.com.
