# Supplementary material for: Care needs and preferences among older adults in an eastern Chinese province between 2013 and 2023: a repeated cross-sectional study
Source: Front Public Health. 2026 Oct 8;14:1939341. doi: 10.3389/fpubh.2026.1939341 (PMC13686901; doi:10.3389/fpubh.2026.1939341)
Supplement: Supplementary file 1 [file Supplementary_file_1.docx]

**Supplemental Material:**

**Title:** Care needs and preferences among older adults in an eastern Chinese province between 2013 and 2023: a repeated cross-sectional study

Supplementary Table S1. Incremental contributions of Andersen model dimensions to care needs and care preferences in the pooled 2018 and 2023 samples

Supplementary Table S2. Multicollinearity diagnostics for the care-need logistic regression models in 2013, 2018 and 2023

Supplementary Table S3. Multicollinearity diagnostics for the care-preference logistic regression models in 2018 and 2023

Supplementary Table S4. Multivariable multinomial logistic regression of factors associated with care preferences in 2018

Supplementary Table S5. Multivariable multinomial logistic regression of factors associated with care preferences in 2023

Supplementary Table S6. Year*disability interaction in the fully adjusted logistic regression model for home-care preference

Supplementary Figure S1. Conceptual framework based on the Andersen Behavioural Model.

**Table S1. Incremental contributions of Andersen model dimensions to care needs and care preferences in the pooled 2018 and 2023 samples**

| Outcome | Model | Block χ² | df | Block *P* | Nagelkerke R² | ΔR² |
| --- | --- | --- | --- | --- | --- | --- |
| Care needs | Model 1 | 2133.848 | 8 | <0.001 | 0.180 | — |
|  | Model 2 | 26.961 | 2 | <0.001 | 0.182 | 0.002 |
|  | Model 3 | 4596.185 | 14 | <0.001 | 0.515 | 0.333 |
| Care preferences | Model 1 | 556.013 | 8 | <0.001 | 0.050 | — |
|  | Model 2 | 17.643 | 2 | <0.001 | 0.051 | 0.001 |
|  | Model 3 | 69.246 | 14 | <0.001 | 0.057 | 0.006 |

**Table S2. Multicollinearity diagnostics for the care-need logistic regression models in 2013, 2018 and 2023**

| Variables | 2013 |  | 2018 |  | 2023 |  |
| --- | --- | --- | --- | --- | --- | --- |
|  | Tolerance | VIF | Tolerance | VIF | Tolerance | VIF |
| **Predisposing factors** |  |  |  |  |  |  |
| Men (ref: Women) | 0.657 | 1.522 | 0.529 | 1.891 | 0.527 | 1.897 |
| Age | 0.646 | 1.547 | 0.671 | 1.491 | 0.678 | 1.474 |
| Unmarried (ref: Married) | 0.994 | 1.006 | 0.990 | 1.010 | 0.989 | 1.011 |
| Widowed | 0.754 | 1.326 | 0.781 | 1.281 | 0.801 | 1.248 |
| Divorced | 0.991 | 1.009 | 0.991 | 1.009 | 0.983 | 1.018 |
| Other | 0.999 | 1.001 | 0.999 | 1.001 | 0.996 | 1.004 |
| Educational level | 0.681 | 1.470 | 0.697 | 1.434 | 0.707 | 1.414 |
| **Enabling factors** |  |  |  |  |  |  |
| Social medical insurance (ref: No) | NA | NA | 0.998 | 1.002 | 0.996 | 1.005 |
| Household income | 0.733 | 1.363 | 0.751 | 1.331 | 0.765 | 1.308 |
| **Need factors** |  |  |  |  |  |  |
| Underweight (ref: Normal weight) | 0.924 | 1.082 | 0.918 | 1.089 | 0.937 | 1.067 |
| Overweight | 0.910 | 1.099 | 0.904 | 1.107 | 0.911 | 1.098 |
| Obesity | 0.944 | 1.060 | 0.931 | 1.074 | 0.932 | 1.073 |
| Health utility | 0.772 | 1.296 | 0.374 | 2.676 | 0.390 | 2.565 |
| Chronic disease | 0.912 | 1.096 | 0.896 | 1.117 | 0.891 | 1.122 |
| Smoking | 0.703 | 1.423 | 0.542 | 1.846 | 0.548 | 1.825 |
| Drinking | 0.764 | 1.309 | 0.719 | 1.392 | 0.776 | 1.289 |
| Physical exercise | 0.864 | 1.157 | 0.874 | 1.145 | 0.888 | 1.126 |
| Health examination (ref: No) | 0.975 | 1.026 | 0.959 | 1.043 | 0.943 | 1.061 |
| Hearing impairment | 0.818 | 1.223 | 0.855 | 1.170 | 0.795 | 1.258 |
| Visual impairment | 0.822 | 1.216 | 0.851 | 1.175 | 0.813 | 1.230 |
| Disability | NA | NA | 0.408 | 2.448 | 0.432 | 2.316 |
| Dementia (ref: No) | NA | NA | 0.880 | 1.136 | 0.893 | 1.120 |
| Social activity (ref: No) | NA | NA | 0.938 | 1.066 | 0.878 | 1.139 |

NA, not assessed in the corresponding survey wave.

VIF, Variance Inflation Factor

**Table S3. Multicollinearity diagnostics for the care-preference logistic regression models in 2018 and 2023**

| Variables | 2018 |  | 2023 |  |
| --- | --- | --- | --- | --- |
|  | Tolerance | VIF | Tolerance | VIF |
| **Predisposing factors** |  |  |  |  |
| Men (ref: Women) | 0.529 | 1.891 | 0.527 | 1.897 |
| Age | 0.671 | 1.491 | 0.678 | 1.474 |
| Unmarried (ref: Married) | 0.990 | 1.01 | 0.989 | 1.011 |
| Widowed | 0.781 | 1.281 | 0.801 | 1.248 |
| Divorced | 0.991 | 1.009 | 0.983 | 1.018 |
| Other | 0.999 | 1.001 | 0.996 | 1.004 |
| Educational level | 0.697 | 1.434 | 0.707 | 1.414 |
| **Enabling factors** |  |  |  |  |
| Social medical insurance (ref: No) | 0.998 | 1.002 | 0.996 | 1.005 |
| Household income | 0.751 | 1.331 | 0.765 | 1.308 |
| **Need factors** |  |  |  |  |
| Underweight (ref: Normal weight) | 0.918 | 1.089 | 0.937 | 1.067 |
| Overweight | 0.904 | 1.107 | 0.911 | 1.098 |
| Obesity | 0.931 | 1.074 | 0.932 | 1.073 |
| Health utility | 0.374 | 2.676 | 0.390 | 2.565 |
| Chronic disease | 0.896 | 1.117 | 0.891 | 1.122 |
| Smoking | 0.542 | 1.846 | 0.548 | 1.825 |
| Drinking | 0.719 | 1.392 | 0.776 | 1.289 |
| Physical exercise | 0.874 | 1.145 | 0.888 | 1.126 |
| Health examination (ref: No) | 0.959 | 1.043 | 0.943 | 1.061 |
| Hearing impairment | 0.855 | 1.170 | 0.795 | 1.258 |
| Visual impairment | 0.851 | 1.175 | 0.813 | 1.230 |
| Disability | 0.408 | 2.448 | 0.432 | 2.316 |
| Dementia (ref: No) | 0.880 | 1.136 | 0.893 | 1.120 |
| Social activity (ref: No) | 0.938 | 1.066 | 0.878 | 1.139 |

NA, not assessed in the corresponding survey wave.

VIF, Variance Inflation Factor

**Table S4. Multivariable multinomial logistic regression of factors associated with care preferences in 2018**

| Variables | Community care vs. Home care | | | Institutional care vs. Home care | | |
| --- | --- | --- | --- | --- | --- | --- |
|  | OR(95%CI) | SE | *P* | OR(95%CI) | SE | *P* |
| **Predisposing factors** |  |  |  |  |  |  |
| Men (ref: Women) | 0.762(0.587,0.987) | 0.133 | 0.040 | 0.968(0.817,1.148) | 0.087 | 0.711 |
| Age | 0.977(0.963,0.991) | 0.007 | 0.001 | 0.962(0.953,0.972) | 0.005 | <0.001 |
| Not married (ref: Married)^a^ | 1.416(1.105,1.814) | 0.127 | 0.006 | 1.289(1.081,1.537) | 0.090 | 0.005 |
| Educational level | 1.150(1.035,1.277) | 0.054 | 0.009 | 1.103(1.026,1.186) | 0.037 | 0.008 |
| **Enabling factors** |  |  |  |  |  |  |
| Social medical insurance (ref: No) | 1.445(0.529,3.943) | 0.512 | 0.473 | 1.297(0.676,2.491) | 0.333 | 0.434 |
| Household income | 1.114(1.028,1.206) | 0.041 | 0.008 | 1.059(1.004,1.118) | 0.028 | 0.037 |
| **Need factors** |  |  |  |  |  |  |
| Underweight (ref: Normal weight) | 1.008(0.670,1.515) | 0.208 | 0.971 | 1.366(1.066,1.751) | 0.127 | 0.014 |
| Overweight | 1.021(0.836,1.246) | 0.102 | 0.839 | 0.817(0.710,0.940) | 0.072 | 0.005 |
| Obesity | 0.825(0.565,1.206) | 0.194 | 0.321 | 0.901(0.707,1.150) | 0.124 | 0.402 |
| Health utility | 0.898(0.760,1.062) | 0.085 | 0.210 | 0.894(0.797,1.003) | 0.059 | 0.056 |
| Chronic disease | 1.138(1.038,1.248) | 0.047 | 0.006 | 1.097(1.029,1.170) | 0.033 | 0.005 |
| Smoking | 1.236(0.929,1.646) | 0.146 | 0.146 | 0.938(0.775,1.135) | 0.097 | 0.510 |
| Drinking | 1.138(0.880,1.472) | 0.131 | 0.324 | 1.156(0.972,1.375) | 0.088 | 0.102 |
| Physical exercise | 1.085(0.977,1.203) | 0.053 | 0.126 | 1.052(0.981,1.128) | 0.036 | 0.158 |
| Health examination (ref: No) | 0.802(0.664,0.969) | 0.096 | 0.022 | 0.994(0.872,1.133) | 0.067 | 0.925 |
| Hearing impairment | 1.038(0.874,1.234) | 0.088 | 0.669 | 1.059(0.942,1.190) | 0.060 | 0.338 |
| Visual impairment | 0.957(0.782,1.172) | 0.103 | 0.673 | 1.016(0.888,1.162) | 0.069 | 0.818 |
| Disability | 0.748(0.517,1.082) | 0.188 | 0.123 | 0.758(0.586,0.980) | 0.131 | 0.034 |
| Dementia (ref: No) | 2.045(1.030,4.060) | 0.350 | 0.041 | 1.609(0.959,2.699) | 0.264 | 0.072 |
| Social activity (ref: No) | 1.179(0.949,1.464) | 0.110 | 0.136 | 1.189(1.027,1.378) | 0.075 | 0.021 |

**Table S5. Multivariable multinomial logistic regression of factors associated with care preferences in 2023**

| Variables | Community care vs. Home care | | | Institutional care vs. Home care | | |
| --- | --- | --- | --- | --- | --- | --- |
|  | OR(95%CI) | SE | *P* | OR(95%CI) | SE | *P* |
| **Predisposing factors** |  |  |  |  |  |  |
| Men (ref: Women) | 0.862(0.625,1.189) | 0.164 | 0.366 | 0.863(0.662,1.124) | 0.135 | 0.274 |
| Age | 0.996(0.978,1.014) | 0.009 | 0.678 | 0.966(0.952,0.980) | 0.007 | <0.001 |
| Not married (ref: Married) | 1.597(1.187,2.148) | 0.151 | 0.002 | 1.991(1.594,2.486) | 0.113 | <0.001 |
| Educational level | 1.356(1.184,1.554) | 0.069 | <0.001 | 1.026(0.920,1.144) | 0.056 | 0.648 |
| **Enabling factors** |  |  |  |  |  |  |
| Social medical insurance (ref: No) | 1.471(0.202,10.699) | 1.012 | 0.703 | 0.979(0.304,3.153) | 0.597 | 0.971 |
| Household income | 1.047(0.948,1.158) | 0.051 | 0.365 | 1.134(1.050,1.225) | 0.039 | 0.001 |
| **Need factors** |  |  |  |  |  |  |
| Underweight (ref: Normal weight) | 1.107(0.641,1.912) | 0.279 | 0.716 | 1.142(0.755,1.725) | 0.211 | 0.530 |
| Overweight | 1.067(0.826,1.377) | 0.130 | 0.620 | 0.826(0.671,1.017) | 0.106 | 0.072 |
| Obesity | 0.849(0.522,1.379) | 0.248 | 0.508 | 1.066(0.767,1.480) | 0.168 | 0.705 |
| Health utility | 0.917(0.743,1.133) | 0.108 | 0.423 | 0.873(0.741,1.028) | 0.084 | 0.104 |
| Chronic disease | 1.112(0.978,1.264) | 0.065 | 0.104 | 1.127(1.021,1.245) | 0.051 | 0.018 |
| Smoking | 1.205(0.842,1.723) | 0.183 | 0.307 | 1.342(1.010,1.783) | 0.145 | 0.042 |
| Drinking | 0.875(0.623,1.229) | 0.173 | 0.441 | 1.036(0.805,1.337) | 0.129 | 0.780 |
| Physical exercise | 1.122(0.976,1.289) | 0.071 | 0.104 | 0.934(0.843,1.035) | 0.053 | 0.194 |
| Health examination (ref: No) | 1.119(0.861,1.454) | 0.134 | 0.401 | 0.980(0.803,1.196) | 0.102 | 0.840 |
| Hearing impairment | 0.818(0.625,1.071) | 0.138 | 0.144 | 0.933(0.764,1.138) | 0.102 | 0.493 |
| Visual impairment | 1.090(0.837,1.418) | 0.135 | 0.524 | 1.107(0.906,1.354) | 0.103 | 0.320 |
| Disability | 1.108(0.770,1.594) | 0.186 | 0.582 | 0.876(0.646,1.187) | 0.155 | 0.392 |
| Dementia (ref: No) | 1.080(0.371,3.144) | 0.545 | 0.888 | 1.727(0.841,3.546) | 0.367 | 0.137 |
| Social activity (ref: No) | 1.224(0.945,1.585) | 0.132 | 0.125 | 1.282(1.047,1.571) | 0.104 | 0.016 |

**Table S6. Year×disability interaction in the fully adjusted logistic regression model for home-care preference**

| Variables | Main-effects model | SE | *P* | Interaction model | SE | *P* |
| --- | --- | --- | --- | --- | --- | --- |
|  | OR(95%CI) |  |  | OR(95%CI) |  |  |
| Year(ref: 2018) | 2.514(2.279,2.773) | 0.050 | <0.001 | 2.586(2.335,2.864) | 0.052 | <0.001 |
| Disability | 1.182(1.007,1.388) | 0.082 | 0.043 | 1.349(1.111,1.638) | 0.099 | 0.002 |
| Year*Disability | — | — | — | 0.752(0.612,0.924) | 0.105 | 0.007 |

**Figure S1. Conceptual framework based on the Andersen Behavioural Model.**
